# “What do you want this information for?”: cognitive interviews with arthritis researchers to inform the development of a health equity-focused demographic survey for outcomes research

**DOI:** 10.1186/s41927-026-00614-2

**Published:** 2026-02-12

**Authors:** Megan M. Thomas, Niki Oveisi, Charlene E. Ronquillo, Mark Harrison, Cheryl Barnabe, Codie Primeau, J. Antonio Avina-Zubieta, Anna Samson, Michael Kuluva, Natasha Trehan, Nikki Bhatti, Ani Methi, Mary A. De Vera

**Affiliations:** 1 The University of British Columbia, Vancouver, BC Canada; 2 Arthritis Research Canada, Vancouver, BC Canada; 3 The University of British Columbia Okanagan, Kelowna, BC Canada; 4 St Paul’s Hospital, Vancouver, BC Canada; 5 University of Calgary, Calgary, AB Canada; 6 Western University, London, ON Canada; 7 Canadian Arthritis Patient Alliance, Ottawa, ON Canada; 8 Creaky Joints, Vancouver, BC Canada; 9 Take a Pain Check, Ottawa, ON Canada

**Keywords:** Arthritis research, Equity, Diversity, Inclusion, Survey

## Abstract

**Background:**

Arthritis research in Canada has historically lacked focus on equity, diversity, and inclusion (EDI), as studies show limited reporting of equity-related factors and underrepresentation of participants who are historically from underserved communities. To address this, the objective of this study was to gather feedback from arthritis researchers in Canada to inform the development of a health equity-focused demographic survey for arthritis outcomes research.

**Methods:**

Cognitive interviews were conducted with individuals who (1) have experience conducting arthritis research; (2) reside in and/or conduct their research in Canada; and (3) speak English or French. A demographic survey developed based on the PROGRESS-Plus framework, which includes characteristics that stratify health opportunities and outcomes (**P**lace of residence; **R**ace, culture, ethnicity, language; **O**ccupation; **G**ender, sex; **R**eligion; **E**ducation; **S**ocioeconomic status; **S**ocial capital; Plus), was iteratively refined through the ‘think-aloud’ technique where participants provided insights and shared perspectives. Thematic analysis was applied to interview transcripts.

**Results:**

Twenty arthritis researchers (11 men) in Canada participated in cognitive interviews. All participants described how the health equity-focused demographic survey was perceived as a feasible and relevant tool. However, some participants expressed concern about questions that focus on perceived sensitive factors (e.g., religion, sexual orientation), while emphasizing there needs to be strong rationale for inclusion to establish patient trust. Three overarching themes were developed: (1) Purpose for collecting demographic data from a health equity lens; (2) Implications of collecting health equity information for data collection, analysis, and interpretation; and (3) Suggestions to enhance survey usability.

**Conclusions:**

A health equity-focused demographic survey developed and refined through cognitive interviews with arthritis researchers may serve as a pragmatic tool to support the integration of EDI in arthritis outcomes research.

**Supplementary Information:**

The online version contains supplementary material available at 10.1186/s41927-026-00614-2.

## Introduction

Equity, diversity, and inclusion (EDI) in research can be understood to refer to appropriate representation, a sense of belonging, and fairness in treatment [1]. Although these principles of EDI have been widely adopted in arthritis care in Canada [2–4], their integration into arthritis research has lagged. Guided by the PROGRESS-Plus framework [5], we previously conducted scoping reviews of clinical trials in rheumatoid arthritis [6], juvenile idiopathic arthritis [7], and systemic lupus erythematosus [8] to explore how EDI has been considered to date in arthritis clinical trials in Canada. The PROGRESS-Plus framework, established by the Campbell and Cochrane Equity Methods Group, describes characteristics that stratify health opportunities and outcomes, and is often used to assess health inequities within populations where disparities often exist (**P**lace of residence; **R**ace, culture, ethnicity, language; **O**ccupation; **G**ender, sex; **R**eligion; **E**ducation; **S**ocioeconomic status (SES); and **S**ocial capital; **P**lus: Personal characteristics associated with discrimination, Features of relationships, and Time-dependent relationships) [5]. In rheumatoid arthritis, we found eight out of 11 demographic populations featured in the PROGRESS-Plus Framework were reported across 42 included studies (place of residence, race, occupation, sex, education, SES, social capital, and age) [6]. However, even when results were reported, there was a lack of standardization with respect to how populations were defined and categorized, making it challenging to compare between studies (e.g., conflating sex and gender terminology). In juvenile idiopathic arthritis, only six population groups’ data were reported (race, sex, education, SES, age, and caregiver education) across five studies [7]. In systemic lupus erythematosus, we found only six population groups had data reported (place of residence, race, sex, SES, social capital, and age) across six studies [8]. As with rheumatoid arthritis, there was non-standard reporting (i.e., terminology conflation), particularly around race and ethnicity. Across the three scoping reviews, sex (often incorrectly reported as gender) and age were the only factors consistently reported in all included studies. In addition to the lack of reporting and standardization, there is a lack of representation for underserved communities in study participants. For rheumatoid arthritis and systemic lupus erythematosus, participants were primarily White, middle-aged females, but in juvenile idiopathic arthritis, they were mostly early adolescent females. This is problematic because factors such as sex and gender, as well as race and ethnicity play a role in the prevalence and incidence of different types of arthritis. For example, rheumatoid arthritis occurs in females almost three times more than in males [9]. Similarly, in systemic lupus erythematosus, the incidence ratio for females is five times higher than that of males, and prevalence nine times higher in females compared to males [10]. Further, in Canada, Indigenous populations are two to three times more likely to have rheumatoid arthritis, and two times more likely to have systemic lupus erythematosus than non-Indigenous populations [11, 12]. In systemic lupus erythematosus the prevalence is also two times higher in Black populations compared to the general population [13]. Thus, health equity factors (e.g., sex, gender, race, ethnicity, etc.) are important to consider, as they are linked to unfair differences in both prevalence and health outcomes for certain populations with arthritis.

A practical implication of the findings from these scoping reviews is the identification of actionable targets to support the incorporation of EDI in arthritis research. Given the identified issues—particularly limited reporting of health equity factors and, when collected, lack of standardized reporting—demographic surveys, which characterize participants in a research study, may serve as a crucial tool to support inclusion in research. There have been efforts across research areas to improve demographic surveys to improve reporting and identify areas for improvement for specific populations, such as LGBTQIA2S + individuals [14]. There have also been efforts to consider underrepresentation and improve diversity in patient and public involvement and engagement in health research within the UK through the development of demographic surveys. These efforts stem from a report by the National Institute for Health and Care Research (NIHR) highlighting that most study participants across research areas are middle-aged, White females [15, 16]. However, to our knowledge, there are no published best practices for demographic surveys in arthritis research, nor demographic surveys with an EDI focus.

The objective of this study was to gather feedback from arthritis researchers in Canada via cognitive interviews to inform the development of a health equity-focused demographic survey for arthritis outcomes research, guided by the PROGRESS-Plus framework.

## Materials and methods

### Study design

This qualitative sub-study was embedded within a larger qualitative study [17] evaluating the experiences and perspectives of arthritis researchers in Canada related to considerations and incorporation of EDI in research, including their own studies. In this sub-study, we conducted cognitive interviews with the same participants to gather feedback on an equity-focused demographic survey developed for arthritis outcomes research, to develop utility before working with patients to enhance the survey’s use. We used the Cognitive Interviewing Reporting Framework [18] to guide the reporting of our findings. We were guided by the pragmatic paradigm which follows the theoretical assumption of real-world applications (rather than holding to a single philosophical stance), allowing for us to have more flexibility when integrating diverse perspectives and methods [19]. This study received institutional ethics approval (BREB# H21-03200), and participants provided informed consent prior to being involved.

### Eligibility criteria and recruitment

Participants were eligible if they: (1) had experience conducting arthritis research studies; (2) resided in and/or conducted their research in Canada; and (3) spoke English or French. Participants were identified using a purposive and respondent driven (snowball) sampling approach [20], and recruitment occurred between May and November 2023. We were purposively sampling for interviewees to achieve diversity in province of residence, sex and gender identity, and racial and ethnic diversity.

### Survey development

Guided by the PROGRESS-Plus framework, an equity-focused demographic survey was initially created by members of the research team (MMT, NO, MADV) to ask questions about each factor in the framework, with the intention of being used to collect information on patient participants at the beginning of an arthritis research study. The survey included 15 questions that map to corresponding PROGRESS-Plus factors (Supplementary Table 1) and was hosted online within the University of British Columbia Qualtrics survey platform. The survey was pilot-tested by five researchers in the same field (e.g., epidemiology, health services research, etc.), who were not involved in the survey development, to establish face validity, which led to the final survey used in this study [21].

### Data collection

Participants were sent a secure link to first provide consent before completing the equity-focused demographic survey. We then followed up with individuals to schedule one-on-one interviews via Zoom. These interviews were structured to first gather arthritis researchers’ perspectives and experiences with incorporating EDI into their research [17], followed by cognitive interviews to collect feedback on the equity-focused demographic survey they had completed as part of this study. Patient research partners (AS, MK, NT, NB) were involved in the conception of the topic guide developed to support the cognitive interviews, including questions and probes aimed at gathering participants’ general impression of the survey, as well as specific feedback on individual questions and response options, and logistical considerations such as administration (Supplementary Table 2). During the cognitive interviews, a combination of think-aloud techniques (i.e., interviewees instructed to report everything that comes to mind for each survey question) and targeted retrospective verbal probing (i.e., interviewees were asked questions with the specific purpose of identifying problems with and their understanding of survey items) were used [18]. We interviewed individuals until data saturation (the degree to which new data repeat what was expressed in previous data) [22] was achieved. All interviews were conducted by MMT, a racialized woman, second-generation immigrant, and graduate student researcher. Participants were informed about the study goal, and the interviewer’s (MMT) role within the project.

### Analysis

We audio-recorded interviews, and transcribed the recordings verbatim using Sonix, an online transcription service (https://sonix.ai), which MMT reviewed for accuracy. We applied thematic analysis according to the following six key steps: (1) Familiarization with the data by transcribing all of the interviews verbatim; (2) Generating initial codes by systematically reviewing the interviews and coding interesting features of the data; (3) Searching for themes across the codes; (4) Reviewing and refining the themes; (5) Defining and naming the themes; and (6) Reporting the themes by presenting them in a table format with a narrative analysis [23]. As researchers’ identities can influence the qualitative research process and results, we state our positionality statements in the supplementary materials for transparency (Supplementary Table 3) [24]. Three members of the research team were the primary analysts of the data: MADV (a woman of colour and epidemiologist), MH (a White man and health economist), and MMT. We used a combination of deductive and inductive coding, organized with NVivo software. This hybrid approach [25] allowed us to begin deductively with developed themes from our prior study [17] and the survey questions using the PROGRESS-Plus framework as a guide, followed by the inductive generation of themes from the data. Crucially, these themes are then combined, mutually enhancing one another. The deductive coding process allowed us to construct the major, or global, themes and the inductive subthemes were constructed within these based on participant responses. Data were analyzed iteratively, (i.e., following each interview, transcripts were analyzed), which allowed us to determine saturation to be the point where no novel codes were identified. Following guidance on cognitive interviews and aligned with the pragmatic paradigm, we used a thematic reduction approach to capture major themes [18, 19, 26] After discussion over several meetings with members of the research team (MADV, MH, and MMT), a coding framework was established. This framework was used by MMT to code all transcripts. Strategies for trustworthiness included reflexive journaling and discussing themes with the research team [27]. Finally, following the analysis, a narrative summary was developed.

## Results

### Participants

Overall, 20 arthritis researchers in Canada took part in cognitive interviews which lasted approximately 15 to 20 min each. Most participants resided in British Columbia at the time of the interview (45%), were White (55%), identified as men (55%), spoke English as their first language (50%), and were married (90%). The complete demographic details of the participants are shown in Table 1.

**Table 1:** Demographic characteristics of participants

Demographic information	Response option	Number (%)
Place of residence*	British Columbia	9 (45%)
	Quebec	4 (20%)
	Ontario	3 (15%)
	Alberta	2 (10%)
	Manitoba	1 (5%)
	Nova Scotia	1 (5%)
Rural or remote setting	No	18 (90%)
	Yes	2 (10%)
Race (select all that apply)	White (e.g. German, Irish, English, Italian, Polish, French, etc.)	11 (55%)
	East Asian (e.g. China, Mongolia, North Korea, South Korea, Japan, Hong Kong, Taiwan, and Macau)	3 (15%)
	South Asian (e.g. Brunei, Burma (Myanmar), Cambodia, Timor-Leste, Indonesia, India, Laos, Malaysia, Pakistan, the Philippines, Singapore, Thailand and Vietnam)	2 (10%)
	West Asian (e.g. Bahrain, Iraq, Iran, Jordan, Kuwait, Lebanon, Oman, State of Palestine, Qatar, Saudi Arabia, Syrian Arab Republic, United Arab Emirates and Yemen)	2 (10%)
	Black, Afro-Caribbean or African American (e.g., Jamaican, Haitian, Nigerian, Ethiopian, Somalian)	1 (5%)
	Hispanic, Latin, or Spanish of origin (e.g., Mexican, Puerto Rican, Cuban, Salvadoran, Dominican, Columbian)	1 (5%)
	Indigenous (e.g., First Nations, Métis or Inuk (Inuit))	1 (5%)
	Central Asian (e.g. Tajikistan, Uzbekistan, Kazakhstan, Turkmenistan, and Kyrgyzstan)	0
	Native Hawaiian or Pacific Islander (e.g., Samoan, Chamorro, Tongan, Fijian)	0
Ethnicity* (select all that apply)	Canadian	9 (45%)
	English	4 (20%)
	Chinese	3 (15%)
	Indian	2 (10%)
	Iranian	2 (10%)
	Irish	2 (10%)
	Catalan	1 (5%)
	German	1 (5%)
	Guadeloupean	1 (5%)
	Italian	1 (5%)
	Ugandan	1 (5%)
First language*	English	10 (50%)
	French	4 (20%)
	Farsi	2 (10%)
	Mandarin	2 (10%)
	Cantonese	1 (5%)
	Catalan	1 (5%)
Language mostly spoken at home* (could list multiple)	English	18 (90%)
	French	2 (10%)
	Mandarin	2 (10%)
Sex	Male	11 (55%)
	Female	9 (45%)
	Intersex	0
	Prefer not to say	0
Current gender identity	Man	11 (55%)
	Woman	9 (45%)
	Non-binary	0
	Indigenous or other cultural gender (e.g., Two-Spirited)	0
	Transgender	0
	Gender fluid	0
	Prefer not to say	0
	Prefer to self describe	0
Sexual orientation	Heterosexual	18 (90%)
	Prefer not to say	2 (10%)
	Aromantic	0
	Asexual	0
	Bisexual	0
	Demisexual	0
	Homosexual	0
	Pansexual	0
Current relationship status	Married	18 (90%)
	Common-law or Co-habiting	1 (5%)
	Separated	1 (5%)
	Dating	0
	Divorced	0
	In a relationship	0
	Polyamorous	0
	Single	0
	Widowed	0
Current religious affiliation	No religious affiliation	9 (45%)
	Christian-Protestant (Anglican, Baptist, etc.)	5 (25%)
	Christian-Catholic	3 (15%)
	Muslim	2 (10%)
	No response	1 (5%)
	Buddhist	0
	Hindu	0
	Indigenous practices	0
	Jewish	0
	Sikh	0
	Other	0
Highest level of education received	Post-graduate degree	19 (95%)
	Graduated a 4-year college, technical school, and/or university	1 (5%)
	Graduated a 2-year college, technical school, and/or university	0
	Attended some college and/or university	0
	Secondary or high school	0
	Elementary, primary or grade school	0
	No schooling completed	0
Current household income (CAD)	$30,000 and under	0
	$30,001-$60,000	0
	$60,001-$90,000	0
	$90,001-$120,000	1 (5%)
	$120,001-$150,000	0
	$150,001-$180,000	0
	$180,001-$210,000	1 (5%)
	$210,001-$240,000	3 (15%)
	$240,001 and over	13 (65%)
	No response	2 (10%)
Current employment	Clinician scientist	14 (70%)
	Researcher	6 (30%)
Career stage	Late (15 + years)	12 (60%)
	Mid (5–15 years)	6 (30%)
	Early (< 5 years)	2 (10%)
Number of members living in household	1	1 (5%)
	2	6 (30%)
	3	2 (10%)
	4	6 (30%)
	5	2 (10%)
	6	2 (10%)
	No response	1 (5%)
Number of dependents	0	5 (25%)
	1	3 (15%)
	2	7 (35%)
	3	2 (10%)
	4	2 (10%)
	5	1 (5%)
Type of inflammatory arthritis studied (select all that apply)	Rheumatoid arthritis	18 (90%)
	Systemic lupus erythematosus	6 (30%)
	Other	6 (30%)
	Ankylosing spondylitis	5 (25%)
	Psoriatic arthritis	5 (25%)
	Gout	3 (15%)
	Juvenile idiopathic arthritis	2 (10%)
**Type of research primarily conducted (select all that apply)**	Observational studies	19 (95%)
	Experimental studies/Clinical trials	10 (50%)
	Qualitative research	5 (25%)
	Health economics research	5 (25%)
	Other	2 (10%)

*Only lists categories with responses or an open text option; all categories can be seen in version 1 of the survey

### Themes

Altogether, when considering how they felt completing the health equity-focused demographic survey, interviewees felt their knowledge of the purpose of the study, as well as their own privilege as researchers in established academic institutions, contributed to their willingness to participate in the cognitive interviews.

*I don’t recall having any concerns about filling it out*,* I was happy to answer all the questions and answer them truthfully. There were none of them that I sort of felt awkward or weird about or uncomfortable answering […] I think I stand on sort of the privileged side of the coin.* -Participant 1

**Fig. 1:**
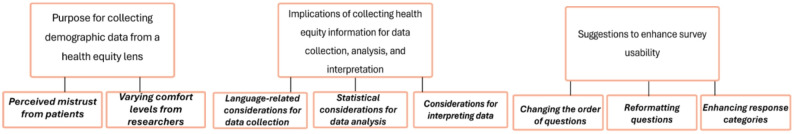
Thematic analysis

\We organized feedback and perspectives of participants about the survey into three overarching themes: (1) Purpose for collecting demographic data from a health equity lens; (2) Implications of collecting health equity information for data collection, analysis, and interpretation; and (3) Suggestions to enhance survey usability. Supporting quotations are provided below and in Table 2 and Fig. 1.

**Table 2:** Additional supporting quotations

Theme	Subtheme	Quotations
Purpose for collecting demographic data from a health equity lens	Perceived mistrust from patients	*“I’m just thinking*,* if I gave that survey to all of my patients*,* it’d be interesting to see the reactions to it. And I think that’s where actually some contextualization is really important when you’re asking questions like that.”* -Participant 4 *“Some people don’t care*,* and some people would want to know why are you asking? So that’s a nice one. You could do that for a few of these questions. Why are we asking?” -*Participant 7 *“I mean one of the ones people might not want*,* they may not want to tell you about their sex lives”.* Participant 15
	Varying comfort levels from researchers when asking potentially sensitive questions	*“So*,* this is like my ignorance. I just don’t know what the right way to go about this*,* […] I think one thing that creates challenges is that some folks get annoyed when they have to answer questions and then they see language that they’re not comfortable with.”-*Participant 2 *“You know*,* people of my generation get a little surprised by the diversity of gender and sexual orientation […]. Sometimes I’m not even sure what it is you know*,* because yeah*,* that’s one of the things I find is changing too quickly.”-* Participant 15 *“I’m probably not the right research background to ask that of. For me these variables come up in* Table 1*as descriptors right*,* I don’t yeah do a lot of deep investigation into these factors*,* so I would defer to others for that.”-*Participant 20
Implications of collecting health equity information for data collection, analysis, and interpretation	Language-related considerations for data collection	*“For example*,* surveys*,* questionnaires*,* you have to have a translator to translate it to another language. Not only language*,* but culturally appropriate questions. […] It requires expertise.”* -Participant 5 *“Language is probably a big issue.”-* Participant 12 *“So*,* if I’m thinking in French. What’s your langue premiere? That actually means what’s your primary language? Which is the one that is most prominent right now. It’s not necessarily the first one that you learned.”* -Participant 14
	Statistical considerations for data analysis	*“So*,* I mean*,* I’ve had 23 and Me and I know I’m half Italian and I’m half Irish*,* English*,* Scottish. So*,* what on earth do I put there? And that’s the point where it starts to feel utterly meaningless.”-* Participant 12 *“You just have to be very precise in the way you ask questions that you get some meaningful data out of it.”-*Participant 18 *“So*,* you’ve got nine categories [for income]*,* and it’s hard to put those into quintiles or quartiles.”-* Participant 19
Suggestions to enhance survey usability	Changing the order of questions	*“People were like either include [sensitive questions] at the end because*,* like*,* people aren’t gonna-they’re gonna click out of the survey.”*-Participant 18 *“You just have to make sure that your survey doesn’t have*,* you know*,* certain required answers to get past that page. Or they’ll just go*,* and they will not do your survey.”-* Participant 19
	Reformatting questions	*“About the rural remote area one […] that definition is not clear to a lot of people […] you want to have a standardized definition”*-Participant 13 *“Actually*,* I would ask it that way. What is the first language that you learned*,* or what is the language that you were fluent in in your childhood?”-* Participant 14
	Enhancing response categories	*“Like I have no clue what pansexual is. Yeah*,* but maybe open ended is good because I do feel like sometimes if people don’t know what this means*,* they might just click something.”-*Participant 2 *“Good*,* because in one of the studies I was telling you about*,* they put they put cis male*,* cis female and half didn’t seem to understand what that meant […] Yeah*,* I like ‘prefer to self-describe’.”*-Participant 9 *“For example*,* education*,* are you really going to see a difference between a two-year and a four-year college? So*,* I think it’s better to get the information perhaps for a fewer number of items.”-* Participant 11

#### Purpose for collecting demographic data from a health equity lens

Participants indicated they routinely collected demographic information for research purposes, especially information related to place of residence, sex, gender, race, and age. However, they highlighted the importance of providing a clear rationale for collecting sensitive information, for example, information related to religion and sexual orientation. Collecting information on religion and sexual orientation (among others) requires considering the perspectives of those responding to the questions, as well as those who are asking the questions. Within this theme, we identified two subthemes: (1) Perceived mistrust from patients, and (2) Varying comfort levels from researchers when asking potentially sensitive questions.

##### Perceived mistrust from patients

Interviewees were mindful that patients might feel reluctant to provide personal information, noting this may be rooted in historical trauma and mistreatment of patients in healthcare and research settings. Participants felt the patients they see in their clinical practice, as well as those involved in previous research, would have concerns about the collection of sensitive demographic survey data. This concern was particularly pertinent among interviewees who were also healthcare providers (i.e., clinician scientists) as they worried asking questions on sensitive factors may unintentionally alienate patients and undermine the patient-provider relationship.

*I’ve kind of wondered when there’s some potentially sensitive questions whether people will feel comfortable answering truthfully and whether they’ll feel […] suspicious or uncomfortable about why we’re asking the question or what the information is for.* -Participant 1

This perception of mistrust was connected to the need to consider potential power imbalances in healthcare and research settings and how patients may feel obligated to participate in studies conducted by their healthcare provider. To combat this concern, individuals spoke to providing explanations in the survey to highlight why certain information is important to collect for factors as captured by this quote from a participant. As one participant explained:

*When you ask these kinds of questions sometimes that are sensitive*,* maybe just explain why we need to capture that*,* right? Why is it important to understand your religion or your sexual orientation? Because*,* you know*,* people don’t just want to give that out away without understanding why.-* Participant 3

Aligned with the need to provide a strong rationale for why certain data are being collected was participants’ perception that it would be unethical to collect sensitive information without a purpose:

*I won’t ever agree to ‘I think [these data] would be nice to have’. When we’re planning a study it’s like no*,* no you have got to have a purpose for that or it’s not ethical to collect that information.-* Participant 12

Interviewee perceptions of mistrust from patients underscored the importance of ensuring that patients understand how their information will be used, along with providing reassurance that researchers will follow ethical policies (e.g., data storage, confidentiality) to protect participant privacy:

*If you have a data leak and then this comes out in the general population*,* and I think that there’s this whole idea of privacy and we have to protect privacy.-*Participant 18

Ultimately, building trust with patients and providing the rationale for why health equity survey data are being collected was perceived as critical for helping patients feel at ease.

##### Varying comfort levels from researchers when asking potentially sensitive questions

Researchers expressed varying levels of comfort with asking potentially sensitive questions. Specifically, there were differing views on the relevance of collecting data on more sensitive equity-related factors. At one end of the spectrum, some participants demonstrated limited understanding of how some equity factors (e.g., sexual orientation) are linked to health outcomes and access to care. Others reflected on their own level of comfort asking patients questions they personally would not want to answer, as shown by the following quote which focused on sexual orientation.

*So why do you need to know that? Why do you need to know my sexual orientation when you’re just interviewing me around EDI? […] The questions are not offensive*,* but kind of like*,* why do question is needed for this survey […] If you think that there is a really good reason for some of the things being collected*,* having an explanation is helpful.* -Participant 8

In contrast, other interviewees emphasized the importance of capturing these factors, namely religion, recognizing their relation to health outcomes and care experiences. These differences in perceptions were linked to participants’ own level of knowledge and experience with considering equity-related factors in research and practice.

*In Hispanic culture in California it is considered insulting to God if you talk about your symptoms. The feeling is in the culture that God only gives you what you can bear. So*,* you can imagine if you’re a practitioner there and patients aren’t talking about how they feel when they come into the office because they feel that they’re doing something to violate their beliefs*,* they’re doing something that would be shameful within their religion*,* that it is indeed a challenge.* -Participant 12

Finally, some participants were concerned that information bias could arise based on researchers’ comfort levels asking sensitive questions, underscoring the need for proper contextualization of these questions:

*And then the issue is by asking those [sensitive] questions*,* are you introducing some bias? Because the people who feel comfortable asking some of those questions maybe may end up being different*,* then you may end up skewing your sample. So*,* I usually find it helpful to have some contextualization to that.-* Participant 4

Overall, understanding researcher levels of comfort was perceived to impact the likelihood of using a health equity-focused demographic survey and asking potentially sensitive questions.

### Implications of collecting health equity information for data collection, analysis, and interpretation

Interviewees also discussed logistical implications of collecting equity-related questions on a demographic survey for a research study with respect to data collection, analysis, and interpretation. This led to identification of 3 subthemes: (1) Language-related considerations for data collection, (2) Statistical considerations for data analysis, and (3) Considerations for interpreting data.

#### Language-related considerations for data collection

A key consideration in collecting this data is language and the potential barriers patients may experience, especially those whose first language is not English. Concerns were raised about how patients may interpret response options to questions they may not necessarily be exposed to or that are not defined in their language(s).

*Yeah*,* I think it would be fine. But my question is that what if there is a patient and they don’t know English? […] it might actually be a challenge with some of these categories*,* for example the gender fluid. What does that mean?*- Participant 5

Another concern related to language was the translation of survey. Participants noted translated items may not capture the true meaning intended by the original survey questions. As this study was based in Canada, where French and English are both official national languages, researchers highlighted the importance of working with experts to ensure French translations truly capture the intended meaning:

*You have to be very careful when you translate these. So*,* for me*,* “langue premiere”*,* which is how I translate first language*,* does not mean the first one that I learned. So*,* I think you need to define it and decide what do you want. I mean*,* I think what you’re getting at*,* is what language do you mostly speak at home?*- Participant 14

Our participants highlighted the necessity to collaborate with content experts and translation services to ensure language barriers are minimized, and the true meaning of questions are preserved. Ultimately, attention to language was perceived as crucial for improving study participant representation and the quality of data collected.

#### Statistical considerations for data analysis

Many interviewees highlighted challenges related to data analysis. Specifically, our participants were concerned about how low cell counts (i.e., too few respondents in a category) may impact study results, particularly for questions with extensive response categories. For example, with ethnicity, participants felt having too many response options made it difficult to analyze the data and draw meaningful conclusions:

*I really struggle with the statistical analysis of all this. […] How on earth do I analyze it? Like with one or two people in each [category]? -*Participant 9

Interviewees also spoke about recategorization to simplify the analysis. For example, grouping similar response options, depending on the purpose of the study, was proposed to address low cell counts:

*Analysis would be really cool. Combining some categories to reduce the number of categories*,* that might be doable. But when collecting data*,* you may want to collect it all […] and then in analysis we can change them.*-Participant 5

Overall, participants felt data analysis could be challenging but emphasized careful planning and consideration about how data are to be analyzed could mitigate potential issues.

#### Considerations for interpreting data

Interviewees discussed how the interpretation of survey results could vary according based on how patients respond. Specifically, some participants wondered whether people’s self-perception of identity can influence how they respond to the questions. It was perceived that there could be distinctions between patient responses that may not capture the true impact on their health or access to healthcare services.

*My nationality is Canadian. This is a nation whose passport I hold*,* you know? Yeah*,* but I might have a Belgian passport because that’s where I was born*,* but I don’t identify as being Belgian […] I think you really want to actually know how does this impact your health?* Participant 17

Further, participants spoke to challenges for patients to interpret the questions, as response options may not accurately and/or fully describe their individual circumstances, which often evolve over time:

*But all these questions you have to ask yourself*,* what does it mean? How many members currently live in your household? What do you mean by members? How many is it? Just how many people live in your house? So*,* last year I had my niece living with us […] But she was- is she a member of my household? I don’t know*,* so you have to be careful.-* Participant 18

Overall, interviewees felt the context around demographic data may be difficult to capture, but important when considering how to interpret findings.

### Suggestions to enhance survey usability

Finally, participants spoke to specific changes they would make if they were to consider administering a survey similar to the one from this study. Proposed changes were grouped into 3 subthemes: (1) Changing the order of questions, (2) Reformatting questions, and (3) Enhancing response categories.

#### Changing the order of questions

Some interviewees suggested questions related to more sensitive topics such as religion, SES, education, and sexual orientation should be moved to the end of the survey. This would ensure that most data are collected even if people decide to stop answering due to discomfort in answering some questions:

*I would put many of the ‘prefer not to say’ questions at the end*,* so that people don’t quit before.-* Participant 15

This perception stemmed from participant experiences collecting more sensitive data in their own research.

*People just didn’t go on*,* so we moved [sensitive questions] to the end and that that helped. […] You can use ‘prefer not to say’*,* but at the end right rather than the beginning.*-Participant 11

Finally, some interviewees felt it could be beneficial to ask patients to provide information they think would be relevant for a researcher or provider to know, rather than it being researcher-driven, with a free-text option at the end of the survey.

*Maybe we need to do a little bit more of ‘What do you think is important for me to know about you?’ […] If we ask people ‘what do we need to know to understand you and to work best with you?’* –Participant 12

#### Reformatting questions

For place of residence, conversations around the difference between rural and remote settings were discussed, as many interviewees highlighted this question should focus on whether patients are able to access the care they need and/or the distance and time they need to travel. It was suggested to separate rural and remote as individual questions.

*You could be rural and maybe you have like really great health care services within your area. That’s not hard to get to […] if we’re asking it in the context of health care*,* sometimes we talk about how far people need to go in order to seek health care.-* Participant 14

There was also a suggestion to add definitions to certain terms within the survey, especially if a free-text response option is not available, to ensure respondents have the same level of understanding when answering questions. Adding definitions was suggested for questions around place of residence, sex, gender identity, and sexual orientation.

*So*,* having maybe like a definition of what it is […] or examples*,* because when I start to hear my kids talking about things*,* I’m like ‘what are you talking about? What is pansexual*,* like*,* what is that?’-* Participant 3

#### Enhancing response categories

Many participants emphasized the need to add a “prefer not to answer” option for questions, as well as open-text options for certain questions including ethnicity, sex, gender, religion, education, SES, and sexual orientation, to prevent offending respondents.

*I think that would be really important to include on a survey like “I prefer not to answer”. You didn’t have that for all of them. Because if you don’t have that option*,* you can make people mad*,* and then they just won’t do the survey.* -Participant 4

For factors that are ordered in nature, such as education and SES, interviewees suggested condensing the response categories to make findings more interpretable, which aligns with the aforementioned subtheme of *Statistical considerations with data analysis.*

*I’m not sure what these [income] levels are based on*,* because you probably won’t use seven or eight or nine levels*,* you know*,* […] you’re probably going to look at above and below the poverty line.-* Participant 20

Interviewees also suggested reordering the response categories for level of education and SES in order of lowest to highest (from top to bottom on the survey page) as a way to help reduce feelings of marginalization.

*Level of education*,* so now you’ve ordered it from highest to lowest […] from an inclusivity point of view that might be not very inclusive to have it in that order.-* Participant 9

The consensus was that for ethnicity, sex, gender, religion, and sexual orientation, the response options should be open-ended, allowing participants to self-describe, even if this may complicate data analysis.

*In some ways I would just keep things open-ended. But this is kind of what we do sometimes when we don’t know how to best measure it. We just put open-ended boxes and then we let folks kind of decide rather than us subscribing categories.-* Participant 2

### Post-interview survey modification

From the cognitive interview data, particularly the final theme, we refined the original health equity-focused demographic survey for use (Supplementary Table 4). The main changes are outlined in Table 3. Changes included reordering of survey questions with questions perceived to be sensitive at the end, recategorization of certain responses as well as changing to open-text response options for several questions, adding a “prefer not to say” response option to most questions, and adding rationale for questions to provide context for why they are being asked.

**Table 3:** Modified demographic survey

Question	Response options	Changes from original survey
In which province or territory do you currently reside?	Alberta	None
	British Columbia	
	Manitoba	
	New Brunswick	
	Newfoundland and Labrador	
	Nova Scotia	
	Ontario	
	Prince Edward Island	
	Quebec	
	Saskatchewan	
	Northwest Territories	
	Nunavut	
	Yukon	
Do you live in a rural setting? * *A rural setting is defined as any area outside of population centers,* with a population less than 1*,*000 people*	Yes	Split rural and remote, added definition of rural
	No	
	Not sure	
Do you live in a remote setting? * *A remote setting is defined as a community that is isolated from other communities and has limited access to services	Yes	Split rural and remote, added definition of remote
	No	
	Not sure	
What is your race (e.g., Black, East Asian, Hispanic, etc.)? ** **Why are we asking this? Race has been identified as a key determinant of health. Racial minorities are at higher risk of experiencing poorer health outcomes and challenges accessing care.	Free text	Made free text response, added rationale for why we are collecting this information
What is your ethnicity (Your cultural identity, chosen or learned from your culture and family. National origin, tribal heritage, religion, language, and culture, can all describe someone’s ethnicity.)? ** **Why are we asking this? Ethnicity can have indirect effects on health outcomes by influencing health beliefs,* the way symptoms are expressed*,* physical functioning*,* entry into health service delivery systems*,* and treatment processes.*	Free text	Made free text response, added rationale for why we are collecting this information
What is the first language you learned?	Free text	Slightly modified the wording of the question to include “learned”
What language do you speak most often at home?	Free text	Slightly modified the wording of the question to include “most often”
What sex* (e.g., male, female, etc.) were you assigned at birth, meaning on your original birth certificate? ** *Sex refers to biological factors **Why are we asking this? Sex is known to effect disease prevalence,* severity*,* and treatment response*	Free text	Made free text response, added a definition and rationale for why we are collecting this information
What is your age range?	< 18 years	Added question
	18–34 years	
	35–54 years	
	55–74 years	
	75–84 years	
	> 85 years	
	Prefer not to say	
Including yourself, how many members currently live in your household?	Free text	Moved to earlier in survey
How many dependents do you have (i.e., a person who relies on you, especially a family member, for financial support)?	Free text	Moved to earlier in survey
What is the highest level of education you’ve completed? ** **Why are we asking this? Education is strongly associated with life expectancy,* morbidity*,* health behaviours*,* and is a strong predictor of one’s socioeconomic status (SES).*	No schooling completed	Moved to earlier in survey, modified categories, ordered from lowest to highest, added a “prefer not to say” option, and added a rationale
	Secondary or high school	
	College, technical school and/or university degree or certificate	
	Post-graduate degree	
	Prefer not to say	
What best describes your current relationship status?	Common-law or Co-habiting	Modified categories
	Dating/in a relationship	
	Divorced/separated	
	Married	
	Polyamorous	
	Single	
	Widowed	
	Prefer not to say	
What is your current household income? ** **Why are we asking this? Income is one of the most important determinants of health and is a strong predictor of one’s socioeconomic status (SES). Level of income shapes overall living conditions,* affects psychological functioning*,* and influences health-related behaviours.*	$30,000 and under	Modified categories, added rationale, added “prefer not to say” option, and moved closer to the end of survey
	$30,001-$65,000	
	$65,001-$100,000	
	$100,001-150,000	
	$150,001 and over	
	Prefer not to say	
What best describes your current gender identity* (e.g., man, woman, non-binary, etc.)? ** *Gender is a socially constructed concept that includes roles,* expectations*,* and behaviors associated with one’s gender identity.* **Why are we asking this? Gender can influence health outcomes and access to care due to different gender norms and behaviours.	Free text	Provided a definition and rationale, made a free-text option, and moved to the end of the survey
What is your sexual orientation*? ** *Sexual orientation describes a person’s emotional,* romantic*,* or sexual attraction to others*,* which can change over time.* **Why are we asking this? Sexual orientation has been shown to impact both mental and physical health,* with sexual minorities experiencing poorer health outcomes overall.*	Free text	Provided a definition and rationale, made a free-text option, and moved to the end of the survey
What is your current religious affiliation? ** **Why are we asking this? Religion can be linked to health outcomes in a variety of ways,* including the provision of social support and community*,* beliefs about diseases and treatment*,* and preferences for care.*	Free text	Provided a rationale, made a free-text option, and moved to the end of the survey
Is there anything else you think we should know about you?	Free text	Added question

*Indicates a definition; ** Indicates a rationale

## Discussion

Guided by the PROGRESS-Plus framework, we gathered feedback from arthritis researchers in Canada to inform the development of a health equity-focused demographic survey for arthritis outcomes research. Our findings highlight the perceived value of using health-equity focused demographic surveys, which could be a vital step toward addressing the EDI limitations identified in arthritis research—specifically, the limited collection of health equity factors and, when such data is collected, the lack of standardized reporting [6–8]. Participants generally agreed demographic surveys with questions on health equity factors provide important information on patient participants which are important when considering EDI in research. However, they emphasized the importance of clearly disclosing the purpose and intent behind collecting sensitive data, such as sexual orientation or religion, to ensure transparency and trust.

Our study highlights an interesting gap between the perceived value of collecting EDI and its implementation. In our prior study [17], participants emphasized the value of prioritizing EDI in research, and agreed there is currently a lack of reporting of equity-related factors. However, when considering the actual collection of these questions in arthritis research, participants showed apprehension about collecting these data. This aligns with existing implementation science research, where despite perceiving value for different concepts or tools, acting toward change remains a challenge [28–30]. Though our survey included questions around factors well-documented to impact health outcomes [5], some participants did not see the relevance, particularly when considering administering the survey for their own research. Participants recognized factors such as sex, gender, race and age impact health outcomes. However, other factors, such as sexual orientation, were not perceived as clearly connected to health outcomes and would require explanation for both patients and researchers/providers. Though perceived as potentially sensitive questions, information on these social determinants of health are well documented to impact patient outcomes [5] and could be important for improving health outcomes and treatment planning in the shared decision-making process between physicians and patients. This perception was also influenced by each researcher’s own comfort levels in answering questions, which could explain whether they would be more or less likely to ask patients these questions. The overarching sentiment from participants was the need to be intentional about collecting these data to ensure the data collected will benefit, rather than exploit, patients. This concern aligns with a prior study with arthritis researchers [17], where participants were concerned that, without intention, research could further increase disparities. This study also found to facilitate actionable change towards EDI in arthritis research, there is a need for community partnerships, diverse research teams, incentives for researchers through funder support, and a sense of humility in research environments. This current study confirms the need for creating safer spaces for researchers to conduct research in [17] to amplify the voices of those who are often missing from arthritis research. Ultimately, though the survey addresses many equity factors, it is important for researchers to use discretion on which factors are most important to collect for their specific research goals, which requires careful consideration. Further, researchers need to balance their intention(s) with participant burden and thus should only collect information which will be purposefully analyzed. Overall, the survey we developed provides a tool for researchers to use to adopt a more comprehensive EDI perspective in research, acting as a starting point in better understanding the impacts of social determinants of health on outcomes for people living with chronic conditions such as arthritis.

The challenges of collecting personal data from patient participants have been recognized across many fields, particularly for chronic diseases because barriers and preferences can change over time, despite reassurances of anonymity, confidentiality, and privacy [31, 32]. A primary concern from participants in this study, as well as from other research [31, 32], is the perception that patients need to understand why certain data are being collected, and how it will be protected. Having strong patient-researcher relationships while providing the rationale supporting the collection of data on equity-related factors could help mitigate any apprehension or mistrust patients may feel towards research and help patients feel more comfortable in sharing personal information. Further, in the process of obtaining consent, researchers need to ensure patient participants fully understand how the information will be collected and used. In a multicultural society like Canada, language and the cultural context of responses can specifically influence how participants understand the collection and use of research data. Language has been reported as a barrier to participation in research in several arthritis studies [33–35], as well as our previous study [17]. When using a demographic survey, such as the one we developed, translation services should be used where possible, and if not, a close family member/friend of a patient should be involved who can help facilitate the translation when informing patients about the opportunity to participate, obtaining consent, and in data collection. It should be noted that though addressing language access is a foundational step, there are still next steps in terms of understanding the cultural context of responses.

This study has limitations. First, we had a small sample for the pilot testing of our demographic survey. As this study was embedded within a previous study, the cognitive interviews themselves did not last more than 15–20 min, however, the survey only had 15 items. As cognitive interviews tend to be more structured [18], we aimed to mitigate this potential limitation by using a combination of deductive and inductive coding to collect both the breadth and depth of information from the transcripts. Additionally, as we interviewed arthritis researchers, the transferability of the survey beyond arthritis may be limited and thus would require further testing. Finally, those who participated in this study likely already have an interest in EDI in arthritis research, which could be a potential selection bias, and could have an impact on findings as our participants may have been more informed on the topic. However, as we were aiming to refine a health equity-focused survey for use in research, it is unlikely that it would be used by individuals who are not interested in considering EDI in any capacity. Therefore, the participants of this study contributed valuable feedback for the use of an equity-focused survey which they themselves could utilize in their own research.

Future research requires consideration of patient perspectives through qualitative research and pilot testing to further modify the demographic survey for use in arthritis studies, as they are the intended participants of the survey. These efforts are underway by our research team; the modified survey is currently being pilot tested with our patient research partners. We will then conduct cognitive interviews with arthritis patients to obtain feedback for further modifications. Though this study was conducted within the context of arthritis, this type of demographic survey is valuable across fields, particularly for other chronic conditions, and could be applied more broadly, which would also require further testing in other settings.

## Conclusion

This study evaluated a heath equity focused demographic survey developed based on the PROGRESS-Plus framework. The survey addresses gaps in data collection, particularly around equity factors that are often missing or underreported in arthritis research in Canada. The findings underscore the potential value of such a tool for improving EDI in research. However, it must first be verified with patients to be administered with intention. There will also need to be careful consideration of the impact on patient participants to ensure it benefits participants without causing harm. Using these survey questions, researchers can begin to better understand how equity factors influence health outcomes, ultimately contributing to a more inclusive approach in arthritis research and a step toward reducing health disparities.

## Supplementary Information

Below is the link to the electronic supplementary material.

Supplementary Material 1

## Data Availability

The qualitative datasets generated and analyzed during the current study are not publicly available due to the inclusion of potentially identifiable information within interview transcripts. Participants did not provide consent for public sharing of these data, and releasing the raw transcripts would violate the conditions of our ethics approval. Therefore, the data are not available upon request.

## Abbreviations

BREB: Behavioural Research Ethics Board
EDI: Equity, diversity, and inclusion
LGBTQIA2S+: Lesbian, Gay, Bisexual, Transgender, Queer, Intersex, Asexual, and Two-Spirit, plus other identities
PROGRESS-Plus: Place of residence; Race, culture, ethnicity, language; Occupation; Gender, sex; Religion; Education; Socioeconomic status; and Social capital; Plus: Personal characteristics associated with discrimination, Features of relationships, and Time-dependent relationships
NIHR: National Institute for Health and Care Research in the UK
SES: Socioeconomic status
