# Supplementary material for: “What do you want this information for?”: cognitive interviews with arthritis researchers to inform the development of a health equity-focused demographic survey for outcomes research
Source: BMC Rheumatol. 2026 Feb 12;10:24. doi: 10.1186/s41927-026-00614-2 (PMC12998049; doi:10.1186/s41927-026-00614-2)
Supplement: Supplementary file 1 — Supplementary Material 1 [file 41927_2026_614_MOESM1_ESM.docx]

Supplementary Table S1. Health equity-focused demographics survey

In order to inform our data analysis, we are aiming to collect different equity, diversity, and inclusion (EDI) factors of participants. This section is guided by the Campbell and Cochrane Equity Methods Group’s PROGRESS-Plus framework for reporting risk factors that lead to inequities in health (<https://methods.cochrane.org/equity/projects/evidence-equity/progress-plus>)

This survey should take you no longer than 10 minutes to complete. Thank you for your interest in our study!

In which province or territory do you currently reside?

- Alberta
- British Columbia
- Manitoba
- New Brunswick
- Newfoundland and Labrador
- Nova Scotia
- Ontario
- Prince Edward Island
- Quebec
- Saskatchewan
- Northwest Territories
- Nunavut
- Yukon

Do you live in a rural and/or remote setting?

- Yes
- No
- Not sure

Which of the following best describes your race? (select all that apply)

- Black, Afro-Caribbean or African American (e.g., Jamaican, Haitian, Nigerian, Ethiopian, Somalian)
- Central Asian (e.g. Tajikistan, Uzbekistan, Kazakhstan, Turkmenistan, and Kyrgyzstan)
- East Asian (e.g. China, Mongolia, North Korea, South Korea, Japan, Hong Kong, Taiwan, and Macau)
- Hispanic, Latin, or Spanish of origin (e.g., Mexican, Puerto Rican, Cuban, Salvadoran, Dominican, Columbian)
- Indigenous (e.g., First Nations, Métis or Inuk (Inuit))
- Native Hawaiian or Pacific Islander (e.g., Samoan, Chamorro, Tongan, Fijian)
- South Asian (e.g. Brunei, Burma (Myanmar), Cambodia, Timor-Leste, Indonesia, India, Laos, Malaysia, Pakistan, the Philippines, Singapore, Thailand and Vietnam)
- West Asian (e.g. Bahrain, Iraq, Iran, Jordan, Kuwait, Lebanon, Oman, State of Palestine, Qatar, Saudi Arabia, Syrian Arab Republic, United Arab Emirates and Yemen)
- White (e.g. German, Irish, English, Italian, Polish, French, etc.)
- Other __________________________________________________

Which ethnicities describes you? Select all that apply.

*Ethnicity is your cultural identity, chosen or learned from your culture and family. National origin, tribal heritage, religion, language, and culture, can all describe someone's ethnicity.*

- Afghan
- Albanian
- Algerian
- American
- Angolan
- Anguillan
- Antiguan
- Argentinian
- Armenian
- Aruban
- Australian
- Austrian
- Azerbaijani
- Azorean
- Bahamian
- Bahraini
- Bangladeshi
- Barbadian
- Belgian
- Belizean
- Beninese
- Bermudian
- Bhutanese
- Bolivian
- Bosnian
- Brazilian
- Bruneian
- Bulgarian
- Burkinabe
- Burmese
- Burundian
- Byelorussian
- Cambodian
- Cameroonian
- Canadian
- Cape Verdean
- Caymanian
- Central African
- Chadian
- Chilean
- Chinese
- Colombian
- Comorian
- Congolese
- Costa Rican
- Croatian
- Cuban
- Cypriot
- Czech
- Danish
- Djiboutian
- Dominica Islander
- Dominican
- Dutch
- Ecuadorian
- Egyptian
- English
- Eritrean
- Estonian
- Ethiopian
- Faroese
- Fijian
- Filipino
- Finnish
- French
- Gabonese
- Gambian
- Georgian
- German
- Ghanaian
- Gibraltarian
- Greek
- Greenlandic
- Grenadian
- Guadeloupean
- Guatemalan
- Guinean
- Guyanese
- Haitian
- Hawaiian
- Honduran
- Hong Konger
- Hungarian
- Icelandic
- Indian (India)
- Indonesian
- Iranian
- Iraqi
- Irish
- Israeli
- Italian
- Ivorian
- Jamaican
- Japanese
- Jordanian
- Kazakh
- Kenyan
- Kittitian/Nevisian
- Korean
- Kosovar
- Kuwaiti
- Kyrgyz
- Laotian
- Latvian
- Lebanese
- Liberian
- Libyan
- Liechtensteiner
- Lithuanian
- Luxembourger
- Macedonian
- Malagasy
- Malawian
- Malaysian
- Malian
- Maltese
- Manx
- Martinican
- Mauritanian
- Mauritian
- Mexican
- Moldovan
- Mongolian
- Montenegrin
- Montserratan
- Moroccan
- Mozambican
- Namibian
- Nepali
- New Zealander
- Nicaraguan
- Nigerian
- Nigerien
- Northern Irish
- Norwegian
- Omani
- Pakistani
- Palestinian
- Panamanian
- Papua New Guinean
- Paraguayan
- Peruvian
- Polish
- Portuguese
- Puerto Rican
- Réunionnais
- Romanian
- Russian
- Rwandan
- Saint Helenian
- Salvadorean
- Samoan
- Saudi Arabian
- Scottish
- Senegalese
- Serbian
- Seychellois
- Sierra Leonean
- Singaporean
- Slovak
- Slovenian
- Somali
- South African
- South Sudanese
- Spanish
- Sri Lankan
- St. Lucian
- Sudanese
- Surinamese
- Swazi
- Swedish
- Swiss
- Syrian
- Tahitian
- Taiwanese
- Tajik
- Tanzanian
- Thai
- Togolese
- Tongan
- Trinidadian/Tobagonian
- Tunisian
- Turkish
- Turkmen
- Ugandan
- Ukrainian
- Uruguayan
- Uzbek
- Venezuelan
- Vietnamese
- Vincentian
- Welsh
- Yemeni
- Zambian
- Zimbabwean
- Abenaki
- Ahousaht
- Algonquin
- Anishinaabe
- Apache
- Assiniboine
- Atikamekw
- Beaver (Dunne-za)
- Blackfoot
- Blood (Kainai)
- Carrier (Dakelh)
- Cayuga
- Chemainus (Stz’uminus)
- Cherokee
- Cheyenne
- Chilcotin (Tsilhqot’in)
- Chipewyan (Denesuline)
- Choctaw
- Coast Salish
- Cowichan
- Cree
- Crow
- Dakota
- Delaware (Lenape)
- Dene
- Dene Tha’ (Slavey)
- Ditidaht
- Dzawada’enuxw
- Ehattesaht
- First Nations
- Gitxsan
- Gwa’sala
- Gwich’in
- Haida
- Haisla
- Halalt
- Hän (Tr’ondëk Hwëch’in)
- Heiltsuk
- Hesquiaht
- Homalco
- Huron (Wendat)
- Huu-ay-aht
- Innu
- Interior Salish
- Inuit
- Inuvialuit
- Iroquois (Haudenosaunee)
- Kaska
- K’omoks
- Ktunaxa (Kutenai)
- Kwakiutl
- Kwakwaﾌｱkaﾌｱﾊｼwakw
- Kyuquot/Cheklesaht
- Laich-kwil-tach
- Lakota
- Lekwungen
- Malahat
- Maliseet
- Mamalilikulla
- Métis
- Mi’kmaq
- Mohawk
- Montagnais
- Moose Cree
- Mowachaht/Muchalaht
- Musqueam
- Nakwaxda’xw
- ’Namgis
- Naskapi
- Navajo
- Nez Perce
- Nisga’a
- Nlaka’pamux (Thompson)
- Nuchatlaht
- Nuu-chah-nulth
- Nuxalk
- Odawa
- Ojibway
- Oji-Cree
- Okanagan (Syilx)
- Oneida
- Onondaga
- Passamaquoddy
- Penelakut
- Piikani
- Plains Cree
- Potawatomi
- Qalipu Mi’kmaq
- Quatsino
- Sahtú (North Slavey)
- Salish
- Saulteaux
- Secwepemc (Shuswap)
- Seneca
- Shawnee
- Shishalh (Sechelt)
- Siksika
- Sioux
- Snuneymuxw
- Squamish
- St’at’imc (Lillooet)
- Stó:lō
- Stoney (Nakoda)
- Swampy Cree
- Tagish
- Tahltan
- Tla’amin (Sliammon)
- Tla-o-qui-aht
- Tlatlasikwala
- Tlicho (Dogrib)
- Tlingit
- Tlowitsis
- Tsek’ene (Sekani)
- Tseshaht
- Tsimshian
- T’Sou-ke
- Tsuu T’ina (Sarcee)
- Tuscarora
- Tutchone
- Ucluelet
- Wet’suwet’en
- Woodland Cree
- W̱SÁNEĆ (Saanich)
- Wuikinuxv

What is your first language?

________________________________________________________________

What language do you mostly speak at home?

________________________________________________________________

What sex were you assigned at birth, meaning on your original birth certificate?

- Male
- Female
- Intersex
- Prefer not to say

What best describes your current gender identity?

- Man
- Woman
- Non-binary
- Indigenous or other cultural gender (e.g., Two-Spirited)
- Transgender
- Gender fluid
- Prefer not to say
- Prefer to self-describe: __________________________________________________

What is your sexual orientation?

- Aromantic
- Asexual
- Bisexual
- Demisexual
- Heterosexual
- Homosexual
- Pansexual
- Prefer not to say

Which best describes your current relationship status?

- Common-law or Co-habiting
- Dating
- Divorced
- In a relationship
- Married
- Polyamorous
- Separated
- Single
- Widowed

What is your current religious affiliation?

- Buddhist
- Christian-Protestant (Anglican, Baptist, etc.)
- Christian-Catholic
- Hindu
- Indigenous practices
- Jewish
- Muslim
- Sikh
- No religious affiliation
- Other: __________________________________________________

What is the highest level of education you’ve completed?

- Post-graduate degree
- Graduated a 4-year college, technical school, and/or university
- Graduated a 2-year college, technical school, and/or university
- Attended some college and/or university
- Secondary or high school
- Elementary, primary or grade school
- No schooling completed

What is your current household income?

- $30,000 and under
- $30,001-$60,000
- $60,001-$90,000
- $90,001-$120,000
- $120,001-$150,000
- $150,001-$180,000
- $180,001-$210,000
- $210,001-$240,000
- $240,001 and over

Including yourself, how many members currently live in your household?

________________________________________________________________

How many dependents do you have? (i.e., a person who relies on you, especially a family member, for financial support)

________________________________________________________________

Display This Question:

If Which of the following best describes your race? (select all that apply) = Indigenous (e.g., First Nations, Métis or Inuk (Inuit))

If yes, are you Two-Spirit?

- Yes
- No

Display This Question:

If Which of the following best describes your race? (select all that apply) = Native Hawaiian or Pacific Islander (e.g., Samoan, Chamorro, Tongan, Fijian)

If yes, are you Māhū?

- Yes
- No

**Supplementary Table S2. Interview guide**

- Introduce self to participant. Ask if they have read the consent form attached to the email, and if they have any questions. If they have not read it, go through it with them prior to starting interview.
- Tell participant the general purpose of the study: to go through a health equity-focused survey they completed in order to collect feedback from arthritis researchers.
- Tell participant that they will be asked to read through the questions and response options to discuss their thoughts and perceptions of using such a survey for arthritis research studies. Remind participant that the interview will be recorded. Remind participant that answers will be used for research, and their names and data collected from the interview will be anonymized.
- Ask permission to begin recording.

**Conduct cognitive interview: show demographic survey and walk through each question and response**

**Ask follow up questions**

How did you feel answering the questions?

*Probe about specific PROGRESS-plus factors (e.g., sexual orientation, religion, etc.)*

What would you change?

*Probe about platform, administering process, understanding of terms*

How would you facilitate the use of this kind of survey in your own research?

*Probe about experiences asking sensitive questions in prior research*

**Before closing:** Is there anything else you would like to add?

**Supplementary Table S3. Modified health equity focused survey**

In order to inform our data analysis, we are aiming to collect different equity, diversity, and inclusion (EDI) factors of participants. This section is guided by the Campbell and Cochrane Equity Methods Group’s PROGRESS-Plus framework for reporting risk factors that lead to inequities in health (<https://methods.cochrane.org/equity/projects/evidence-equity/progress-plus>)

This survey should take you no longer than 10 minutes to complete. Thank you for your interest in our study!

In which province or territory do you currently reside?

- Alberta
- British Columbia
- Manitoba
- New Brunswick
- Newfoundland and Labrador
- Nova Scotia
- Ontario
- Prince Edward Island
- Quebec
- Saskatchewan
- Northwest Territories
- Nunavut
- Yukon

 Do you live in a rural setting? *

**A rural setting is defined as any area outside of population centers, with a population less than 1,000 people*

- Yes
- No
- Not sure

Do you live in a remote setting? *

**A remote setting is defined as a community that is isolated from other communities and has limited access to services any area outside of population centers*

- Yes
- No
- Not sure

What is your race (e.g., Black, East Asian, Hispanic, etc.)? List all that apply **

***Why are we asking this? Race has been identified as a key determinant of health. Racial minorities are at higher risk of experiencing poorer health outcomes and challenges accessing care.*

________________________________________________________________

What is your ethnicity (Your cultural identity, chosen or learned from your culture and family. National origin, tribal heritage, religion, language, and culture, can all describe someone's ethnicity.)? List all that apply**

***Why are we asking this? Ethnicity can have indirect effects on health outcomes by influencing health beliefs, the way symptoms are expressed, physical functioning, entry into health service delivery systems, and treatment processes.*

________________________________________________________________

What is the first language you learned?

________________________________________________________________

What language do you speak most often at home?

________________________________________________________________

What sex* (e.g., male, female, etc.) were you assigned at birth, meaning on your original birth certificate? **

**Sex refers to biological factors*

***Why are we asking this? Sex is known to effect disease prevalence, severity, and treatment response*

________________________________________________________________

What is your age range?

- <18 years
- 18-34 years
- 35-54 years
- 55-74 years
- 75-84 years
- >85 years
- Prefer not to say

Including yourself, how many members currently live in your household?

________________________________________________________________

How many dependents do you have? (i.e., a person who relies on you, especially a family member, for financial support)

________________________________________________________________

What is the highest level of education you’ve completed? **

***Why are we asking this? Education is strongly associated with life expectancy, morbidity, health behaviours, and is a strong predictor of one’s socioeconomic status (SES).*

- No schooling completed
- College, technical school and/or university degree or certificate
- Post-graduate degree
- Prefer not to say

Which best describes your current relationship status?

- Common-law or Co-habiting
- Dating/in a relationship
- Divorced/separated
- Married
- Polyamorous
- Single
- Widowed
- Prefer not to say

What is your current household income? **

***Why are we asking this? Income is one of the most important determinants of health and is a strong predictor of one’s socioeconomic status (SES). Level of income shapes overall living conditions, affects psychological functioning, and influences health-related behaviours.*

- $30,000 and under
- $30,001-$65,000
- $65,001-$100,000
- $100,001-$150,000
- $150,001 and over
- Prefer not to say

What best describes your current gender identity* (e.g., man, woman, non-binary, etc.)? **

**Gender is a socially constructed concept that includes roles, expectations, and behaviors associated with one’s gender identity.*

***Why are we asking this? Gender can influence health outcomes and access to care due to different gender norms and behaviours*

__________________________________________________

What is your sexual orientation*? **

**Sexual orientation describes a person's emotional, romantic, or sexual attraction to others, which can change over time.*

***Why are we asking this? Sexual orientation has been shown to impact both mental and physical health, with sexual minorities experiencing poorer health outcomes overall.*

__________________________________________________

What is your current religious affiliation? **

***Why are we asking this? Religion can be linked to health outcomes in a variety of ways, including the provision of social support and community, beliefs about diseases and treatment, and preferences for care.*

__________________________________________________

Is there anything else you think we should know about you?

__________________________________________________
